# Identification of *Lactobacillus* proteins with different recognition patterns between immune rabbit sera and nonimmune mice or human sera

**DOI:** 10.1186/s12866-016-0631-9

**Published:** 2016-02-09

**Authors:** Sabina Górska, Barbara Buda, Ewa Brzozowska, Martin Schwarzer, Dagmar Srutkova, Hana Kozakova, Andrzej Gamian

**Affiliations:** Department of Medical Microbiology, Ludwik Hirszfeld Institute of Immunology and Experimental Therapy of the Polish Academy of Sciences, Wroclaw, Poland; Department of Animal Products Technology and Quality Management, Wroclaw University of Environmental and Life Sciences, Faculty of Food Science, Wroclaw, Poland; Institute of Microbiology, Laboratory of Gnotobiology, Academy of Sciences of the Czech Republic v. v. i., 549 22 Novy Hradek, Czech Republic

**Keywords:** *Lactobacillus*, Probiotics, Surface proteins, Immunoreactivity, Sera

## Abstract

**Background:**

The genus *Lactobacillus* belongs to a large heterogeneous group of low G + C Gram-positive anaerobic bacteria, which are frequently used as probiotics. The health-beneficial effects, in particular the immunomodulation effect, of probiotics depend on the strain and dose used. Strain variations may be related to diversity of the cell surface architecture of bacteria and the ability to express specific antigens or secrete compounds. The use of *Lactobacillus* as probiotic requires a comprehensive understanding of its effect on host immune system. To evaluate the potential immunoreactive properties of proteins isolated from four *Lactobacillus* strains: *L. johnsonii* 142 and *L. johnsonii* 151, *L. rhamnosus* LOCK 0900 and *L. casei* LOCK 0919, the polyclonal sera obtained from mouse and human have been tested as well as with sera from rabbits immunized with whole lactobacilli cells.

**Results:**

The reactivity of isolated proteins detected by SDS-PAGE and Western blotting was heterogeneous and varied between different serum samples. The proteins with the highest immunoreactivity were isolated, purified and sequenced, in particular the fractions were identified as phosphoglycerate kinase (*L. johnsonii* 142), glyceraldehyde 3-phosphate dehydrogenase (*L. johnosnii* 142, *L. rhamnosus* LOCK 0900), hypothetic protein JDM1_1307 (*L. johnsonii* 151) and fructose/tagatose-bisphosphate-aldolase (*L. casei* LOCK 0919).

**Conclusion:**

The different prevalence of reactions against tested antigens in rabbit, mouse and human sera may indicate significant differences in immune system and commensal cross-talk in these groups. The identification of immunoreactive lactobacilli proteins opens the possibility to use them as an antigens for development of vaccines.

## Background

*Lactobacillus* are nonpathogenic Gram-positive bacteria that form part of the normal intestinal microbiome in humans and animals. They are a large group of bacteria growing under microaerophilic conditions for optimal growth temperature in the range of 30–40 °C and pH 5.5–6.2 typically. The *Lactobacillus* species are commonly used to manufacture fermented milk products and some of them are considered probiotics on account of their health benefits [1–5]. Moreover, they are “generally regarded as safe” according to The American Food and Drug Administration due to their long history of safe use in fermented foods and their presence in the normal intestinal and urogenital microbiome of humans. The mechanisms underlying probiotic effect are generally attributed to the interaction of probiotics with other microorganisms (bacteria-bacteria cross-talk) or with host cells (bacteria-host cross-talk). The interactions depend on the viability of probiotic cells, since it is exerted by competitive exclusion, direct inhibition of certain microorganisms involving production of antimicrobial molecules or increased growth of healthy components of the microbiota, while the interaction with the host is based on the capacity of host cells to recognize specific bacterial components or products, giving rise to response that commonly involve the immune system [6].

The lactic acid bacteria influence has been described as enhancement of non-specific immunity as well as adjuvant and immunoregulatory effects in adaptive immune responses. However, the bacterial components responsible for these effects are often left unidentified. The *Lactobacillus* bacteria show great diversity in the cell surface architecture which may influence to the physicochemical properties of the bacterial cell and strain specific properties. The cell envelope of lactobacilli is composed of the bilayer lipid cell membrane with embedded proteins anchored to the cell wall and covered by thick multilayered peptidoglycan together with lipo- and teichoic acids, pili and polysaccharides sheet. Polysaccharides can either attach to the cell surface like an outer capsule and often be covalently bound to N-acetylmuramic acid of peptidoglycan or be loosely attached with it or be secreted to the surrounding environment.

The cell surface proteins are anchored to the cell wall by various mechanisms or released into the surrounding medium, where they reassociate with the cell wall through electrostatic interactions [7]. Covalently anchored proteins can be divided into N- or C-terminally anchored proteins, lipoproteins and LPXTG-anchored proteins (or sortase-dependent proteins). N-terminally anchored proteins are involved in extracellular transport, signal transduction and protein turnover, while lipid-anchored proteins are involved in adhesion, antibiotic resistance, folding and translocation of proteins [8–10]. LPXTG-anchored proteins have been demonstrated to be crucial for the interactions of pathogenic and nonpathogenic bacteria with their hosts [11]. Some species of lactobacilli produce an additional paracrystalline layer of proteins called as S-layer. This is a two-dimensional crystal layer consisting of 25–71 kDa molecular mass proteins mainly and highly predicted overall p*I* value (9.4–10.4), and represent 10–15 % of the total cell protein content [12–14]. The lattice symmetry of *Lactobacillus* S-layer proteins is of oblique or hexagonal type [15]. The most often proposed function for S-layer is mainly reduction the ability of pathogens adhesion and invasion or reduction theirs toxins activity [4, 5, 16–19]. Some other cell surface proteins were also shown to be involved in adhesion to human intestinal cells and mucins, stimulation of cytokine secretion and mediation of co-aggregation of probiotic bacteria with pathogenic bacteria [20]. Pili have been identified at the genome level in some lactobacilli, however only in *L. rhamnosus* GG polymeric structures and functionality have been characterized [21]. The flagella have been recognized only in twelve species of lactobacilli, and comprises flagellin, protein which is suggested to activate of signaling pathways and modulate of host immune cells [22].

The other key molecules are extracellular proteins which could regulate certain signaling pathways and cellular responses, including secretion of different effector molecules such chemokines, cytokines, antibacterial peptides, mucus secretion, induction of changes in the surface properties and modulation of the immune function and the immune response of the host cells [23]. It is known that *Lactobacillus* bacteria interact with epithelial cells trough several mechanisms mediated by extracellular and cell-surface-associated proteins that bind to mucus and intestinal cells [24]. *Lactobacillus* adhesion to mucus involves mucus binding proteins (Mubs) which in addition to the same domain organization typical for cell surface proteins share a mucus binding domain [25]. The MucBPs (putative mucus binding proteins) have been found among 13 different *Lactobacillus* strains [26]. *L. fermentum* produced a 32 kDa surface-associated protein (32-mMubp) that is suggested to mediate adhesion to mucus [27], whereas studies with *L. rhamnousus* GG have shown a mucus binding factor with a presumed ancillary involvement in pilus-meditative mucosal adhesion [28]. Recent studies of *L. casei* BL23 sortase have shown that this sortase might be involved in adhesion of this strain to Caco-2 and HT cells [29]. *L. johnsonii* has been found to produce elongation factor Tu (EF-Tu) which mediated the attachment to human intestinal cells and mucins [20]. The issue of lactobacilli antigens supporting probiotic action in probiotic bacteria has been extensively reviewed by Lebeer et al. [30, 31], by Kleerebezem et al. [10] and by Sengupta et al. [32], however studies on the immunoreactivity of the lactobacilli molecules are sparse. In our previous studies of two structurally different exopolysaccharides isolated from *L. johnsonii* 142 and *L. johnsonii* 151 [33, 34] we have shown that both antigens differed in their reactivity with human sera. It appeared that physiological sera of healthy adult blood donors contain antibodies reactive with studied exopolysaccharides, at relatively high titers. Moreover, such antibodies are also present in human umbilical cord blood sera.

The aim of this study was to identify immunoreactive proteins of four *Lactobacillus* strains: *L. johnsonii* 142 and *L. johnsonii* 151*, L. rhamnosus* LOCK 0900 and *L. casei* LOCK 0919. The proteins immunoreactivity with 13 sera (from mice, from human healthy adults, human umbilical cord blood sera and from rabbits immunized with cells) was detected by SDS-PAGE and Western blotting. As a result, several major immunoreactive proteins were identified.

## Results

**Fig. 1:**
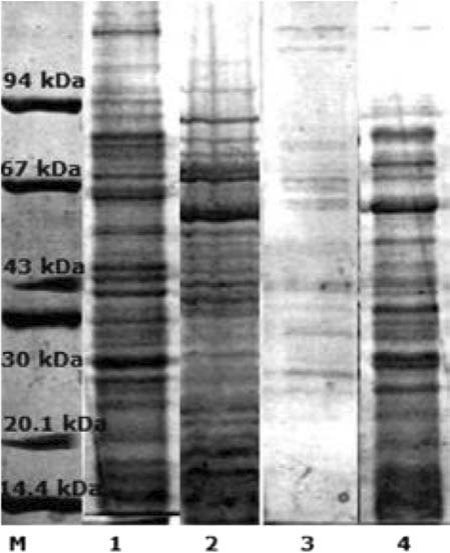
SDS-PAGE profile with Coomassie Brilliant Blue staining of proteins isolated from *Lactobacillus* strains: M – low molecular mass marker, 1 – *L. johnsonii* 142, 2 – *L. johnsonii* 151, 3 – *L. rhamnosus* LOCK 0900, 4 – *L. casei* LOCK 0919. These images were cut from the same gel, merged and the appropriate scaling was used

The protein concentration in extracts of the studied strains was significantly different and varied, ranging from 3.1 to 18.7 mg/ mL. We observed that extraction of protein with sodium dodecyl sulfate procedure was no effective in case of *L. rhamnosus* LOCK900, probably due to the presence of polysaccharide slime produced by these bacteria. Thus the slime has been removed mechanically which allowed to increase the efficiency of the protein extraction by 300 %. Samples were electrophoresed and stained with Coomassie Brilliant Blue. The representative results are shown in Fig. 1. It could be demonstrated that proteins ranged in molecular mass from > 100 kDa to about 20 kDa. The profiles were quite variable, emphasizing the heterogeneity in species, however it has been shown to share several predominant bands.

**Fig. 2:**
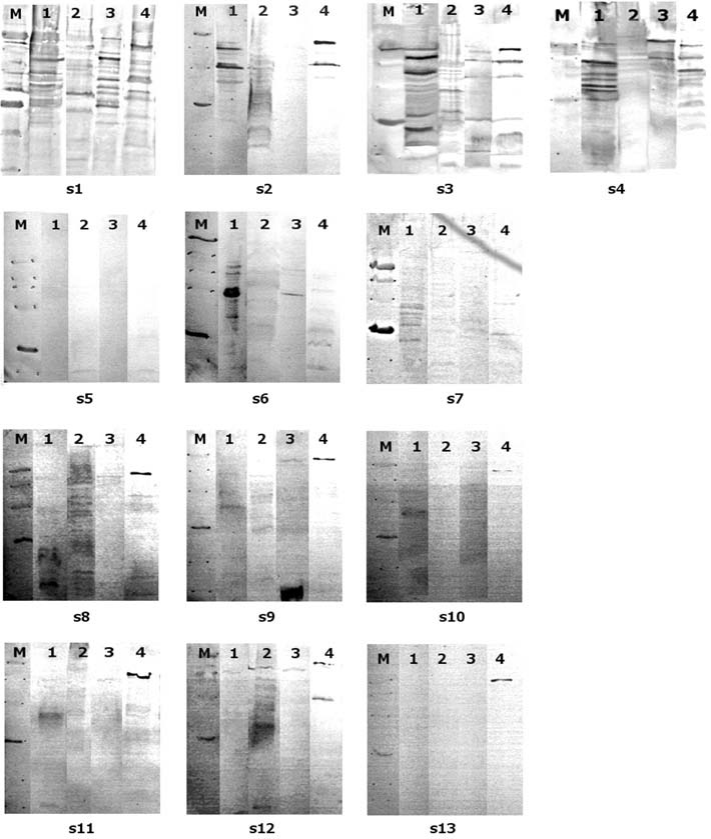
Immunoblotting of proteins isolated from analyzed strains: M – low molecular mass marker: 94, 67, 43, 30, 20.1, 14.4 kDa, line 1 – *L. johnsonii* 142, line 2 – *L. johnsonii* 151, line 3 – *L. rhamnosus* LOCK 0900, line 4 – *L. casei* LOCK 0919, with sera: s1– rabbit polyclonal serum against *L. paracasei* LOCK 0912, s2 – rabbit polyclonal serum against *L. animalis murinus* 148, s3 – rabbit polyclonal serum against *L. johnsonii* 142, s4 – rabbit polyclonal serum against *L. johnsonii* 151, s5 – serum from germ free mouse, s6 – serum from mouse living under pathogen free conditions, s7 – serum from mouse living under conventional conditions, s8-s10 sera from human healthy adults, s11-s13 human umbilical cord blood sera. These images were cut from the same gel, merged and the appropriate scaling was used

For the analysis of immunoreactivity, protein samples were transferred to a polyvinylidene difluoride membrane for immunoblotting. This experiment resulted in a detection of proteins reacting with analyzed sera and several delineations were revealed. In case of *L. johnsonii* 142, there were five protein bands with the molecular mass of about 90, 67, 50, 25 and 18 kDa reacting with homologous sera (Fig. 2, s3, line 1), two immunoreactive protein fractions reacting with sera against *L. animalis murinus* 148 (ca. 67 and 43 kDa, Fig. 2, s2, line 1), six major protein fractions reacting with sera against *L. johnsonii* 151 (ca. 90, 67, 53, 43, 38 and 35 kDa, Fig. 2, s4, line 1) and several proteins ca. 67, 43, 30 kDa reacting with sera against *L. paracasei* LOCK 0912 (Fig. 2, s1, line 1). We detected a marked reaction of protein about 30 kDa with sera from mouse kept under specific-pathogen-free conditions (SPF conditions, Fig. 2, s6, line 1) and with mouse kept under conventional conditions (Fig. 2, s7, line 1), however there were no reactions with sera obtained from germ free mouse (Fig. 2, s5, line 1). The antibodies against protein 35 kDa were detected in several human sera, namely s8-s10 and one umbilical cord blood serum (s11), but the reaction was very weak. In case of protein extract from *L. johnsonii* 151 we observed several immunoreactive proteins, however homologous reactivity was blurred (Fig. 2, s4, line 2) and enabling determination of the approximate molecular mass. Strain 151 produced small proteins (ca 40 kDa) which reacted with other rabbit sera (Fig. 2, s1-s3, line 2), and very weak with human sera (Fig. 2, s8-s13, line 2). We didn’t observe reactivity with sera from mice (Fig. 2, s5-s7, line 2). Considering human origin species *L. casei* LOCK 0919 we observed a broad reactivity of protein with molecular mass of about 90 kDa, in particular, as the only one of the tested bacterial proteins reacted clearly with human umbilical cord blood sera (Fig. 2, s8-s10, line 4). Weak cross-reactivity with mouse serum has been noticed for proteins of molecular mass of 30 and 43 kDa isolated from LOCK 0900 (Fig. 2, s7, line 4). Interestingly, six proteins of LOCK 0900 and LOCK 0919 have been shown to cross-react with serum against *L. johnsonii* 142 (Fig. 2, s3, lines 3–4).

**Fig. 3:**
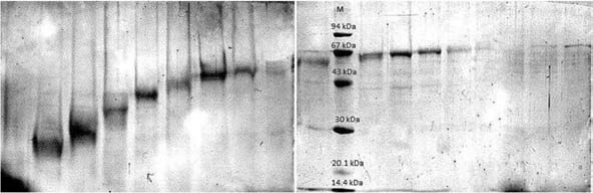
The SDS-PAGE profile of a separated proteins isolated from *Lactobacillus johnsonii* 151 in the presence of low molecular mass marker – 94, 67, 43, 30, 20.1, 14.4 kDa by continuous-elution electrophoresis (Prep-Cell apparatus Model 491 Bio-Rad). Samples are electrophoresed through a cylindrical gel. As molecules migrate through the gel matrix, they separate into bands. Individual bands migrate off the bottom of the gel where they pass directly into the patented elution chamber for collection. The resulting liquid fractions (2 ml) were pooled (5 fractions), dried and analyzed on SDS-PAGE. Gels were stained with Coomassie Brilliant Blue

**Fig. 4:**
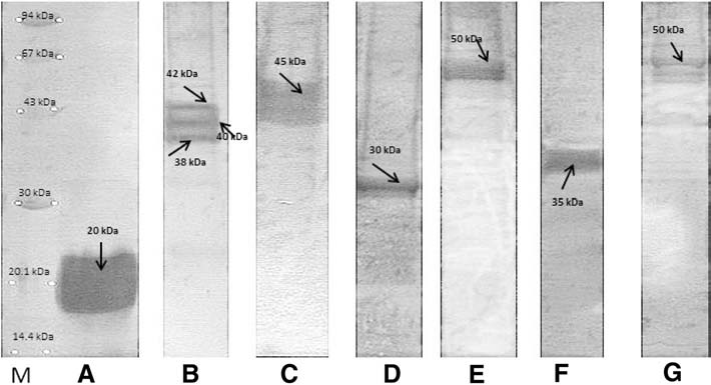
The selected immunoblots of separated proteins: A – immunoreactive protein of *L. johnsonii* 142 reacting with homologous serum (s3), B – immunoreactive proteins of *L. johnsonii* 142 reacting with anti-*L. paracasei* LOCK 0912 (s1), C – immunoreactive protein of *L. johnsonii* 151 reacting with anti-*L. johnsonii* 151 (s4), D – immunoreactive proteins of *L. rhamnosus* LOCK 0900 reacting with anti-*L. paracasei* LOCK0912 (s1), E – immunoreactive proteins of *L. rhamnosus* LOCK 0900 reacting with human cord blood sera (s12), F – immunoreactive proteins *L. casei* LOCK 0919 reacting with mouse CV sera (s6), G – immunoreactive proteins *L. casei* LOCK 0919 reacting with rabbit anti-*L. paracasei* LOCK 0912 (s1). These images were taken from different blots, merged and the appropriate scaling was used

To identify the most immunogenic/immunoreactive proteins, electrophoretic preparation of isolated proteins from four analyzed strains was made using Prep-Cell Apparatus (491 model Bio-Rad) and re-analyzed in the immunoblotting assays. The representative results were shown on Figs. 3 and 4. Bands with molecular mass of about 20 kDa (immunoreactive with serum against *L. johnsonii* 142) and 38, 40, 42 kDa (reacting with rabbit, human or mouse sera) isolated from *L. johnsonii* 142, one spot of 45 kDa from *L. johnsonii* 151 (reacting with rabbit sera against *L. johnsonii* 151 and human sera), and two bands of 35 and 50 kDa from *L. rhamnousus* LOCK 0900 and also two bands of 30 and 50 kDa isolated from *L. casei* LOCK 0919 showing strong immunoreactivity features were cut from the gel and analyzed in LC-MS/MS. Proteins were identified by comparative analysis of peptides masses (NCBI, UniProt databases) using MASCOT and statistical analysis. These procedures failed to obtain and identify the most reactive protein (90 kDa) in extract of LOCK 0919. The results of sequencing and most like homologies of isolated and analyzed proteins with spectrometric method have been summarized in Table 1. The highest protein sequence coverage of *L. johnsonii* 142 we observed for spot with molecular mass about 42 kDa. This protein has been identified as the phosphoglycerate kinase (79 % protein sequence coverage), whereas the protein 45 kDa of *L. johnsonii* 151 strain has not been identified. However, we noticed low sequence coverage (39 %) corresponded to hypothetical protein JDM1_1307 from *L. plantarum* JDM1. Other immunoreactive proteins of *L. johnsonii* 142 were identified as glyceraldehyde 3-phosphate, dehydrogenase 30S ribosomal protein S7 and surface antigen NLP/P60 with 67, 60 and 48 % protein sequence coverage, respectively. Spot with molecular mass of 35 kDa isolated from strain LOCK 0900 was identified as glyceraldehyde 3-phospahte (60 %), however a slightly lower sequence coverage (59 %) was obtained for fructose/tagatose-bisphosphate-aldolase (59 %). The 50 kDa spot of LOCK 0900 was characterized by a low homology with aminopeptidase C (31 %). Interestingly, the immunoreactive spot with molecular mass around 30 kDa isolated from *L. casei* LOCK 0919 was identified as fructose/tagatose-bisphosphate-aldolase (88 % protein sequence coverage) while for the second analyzed protein (50 kDa) lower score (42 %) for glucose-6-phosphate isomerase was obtained.

**Table 1:** Identification of immunoreactive proteins isolated from *Lactobacillus* strain

Strain number	Estimated molecular mass of isolated protein [kDa]	Homologous protein name	Nominal molecular mass of homologous proteins [kDa]	Origin of homologous protein	Decription	Homology [%]
*L. johnsonii* 142	20	30S ribosomal protein S7 (gi׀116495953)	17,912	*Lactobacillus casei* ATCC 334	It is directly linked to the 16S rRNA	60
	38	Glyceraldehyde 3-phosphate dehydrogenase (GAPDH) (gi׀199597272)	36,929	*Lactobacillus rhamnosus* HN001	It is involved in the glucose metabolism and has oxidoreductase activity	67
	40	surface antigen NLP/P60 (gi ׀199598074)	40,947	*Lactobacillus rhamnosus* GG	Human mucus binding proteinn	48
	42	phosphoglycerate kinase (gi ׀199597273)	42,187	*Lactobacillus rhamnosus* HN001	It is involved in glycolysis. Transfers a phosphate group from 1,3-bisfosfoglicerynianu to ADP creating 3-phosphoglycerate and ATP.	79
*L. johnsonii* 151	45	Hypothetical protein JDM1_1307 (gi ׀254556474)	44,729	*Lactobacillus plantarum* JDM1	Function unknown	39
*L. rhamnosus* LOCK 0900	35	Glyceraldehyde 3-phosphate dehydrogenase (GAPDH) (gi׀ 116494473)	36,912	*Lactobacillus casei* ATCC 334	It is involved in the glucose metabolism and has oxidoreductase activity	60
		Fructose/tagatose-bisphosphate-aldolase (gi׀ 229551479)	36,360	*Lactobacillus rhamnosus* LMS2-1	It is involved in the metabolism of carbohydrates. It also participates in the binding of zinc ions, and its activity is stimulated by some bivalent ions	59
	50	Aminopeptidase C (gi׀258509341)	50,737	*Lactobacillus rhamnosus* GG	It is involved in proteolysis	31
*L. casei* LOCK 0919	30	Fructose/Tagatose-bisphosphate-aldolase (gi׀ 116493996)	31,640	*Lactobacillus casei* ATCC 334	It is involved in the metabolism of carbohydrates. It also participates in the binding of zinc ions, and its activity is stimulated by some bivalent ions	88
	50	Glucose-6-phosphate isomerase (gi׀ 116494625)	49,292	*Lactobacillus casei* ATCC 334	Catalyze the reaction: transformation of D-glucose-6-phosphate into D-fructose-6-phosphate	42

## Discussion

The unraveling of the molecular mechanisms underlying the lactobacilli biological effects is an attractive field for investigation. Among the different cellular molecules, proteins might mediate certain interactions, since they would be able to interact directly with host immune system. To date, our knowledge of the identity of proteins responsible for immunomodulatory effect is very limited, although several studies have reported the interaction between lactobacilli proteins and human cells, few have been identified and characterized so far [35–37]. In our studies we focused on identification of potentially immunoreactive/immunogenic proteins of four strains: two isolated from mouse (*L. johnsonii* 142 and 151) and two isolated from human (*L. rhamnosus* LOCK 0900 and *L. casei* LOCK 0919) using electrophoretic, immunoblotting and mass spectrometry method. The reactivity of isolated proteins detected by SDS-PAGE and immunoblotting was heterogeneous and varied between different serum samples. The different prevalence of reactions against tested antigens in rabbit, mouse and human sera may indicate significant differences in immune system and commensal cross-talk in these group. The reason could be the way of immunization, as rabbit vaccination differs from a native intestinal stimulation of immunity, moreover rabbit immune response differs markedly from that of mice and human organism. Lactobacilli as food-grade and potentially probiotic organisms with therapeutically purposes should be non-immunogenic reflected in an inability to evoke antibody responses against themselves. However it has been shown that some *Lactobacilli* and *Bifidobacteria* strains can stimulate the production of secretory antibodies such as IgA and IgG. Maassen et a. described that orally administered individual *Lactobacillus* strains are able to differentially affect IgG1 versus IgG2a antibody, depending on bacteria growth phase [38]. Prangli et al. found IgG antibodies against common lactobacilli cytoplasmic proteins GroEl, enolase, transcription factor EF-Ts and EF-Tu in children with type 1 diabetes and coeliac disease [39]. In our study we also indicate immunoreactive proteins which are recognized by rabbit/mouse and human immune system and elicit an antibodies. The proteins with the highest immunoreactivity were isolated, purified and sequenced, in particular the fractions were identified as phosphoglycerate kinase, 30S ribosomal protein, surface antigen NLP/P60 (*L. johnsonii* 142), glyceraldehyde 3-phosphate dehydrogenase (*L. johnosnii* 142, *L. rhamnosus* LOCK 0900), hypothetic protein JDM1_1307 (*L. johnosnii* 151), fructose/tagatose-bisphosphate-aldolase (*L. casei* LOCK 0919 and *L. rhamnosus* LOCK 0900) and glucose-6-phosphate isomerase (*L. casei* LOCK 0919) and aminopeptidase C (*L. rhamnosus* LOCK 0900). This protein are well-known as cytosolic metabolic enzymes or translation protein and not as a surface proteins. However, over the past decade, it has become clear that many proteins have one or more unique functions over-and-above the principal biological action of the specific protein. This phenomenon is now known as protein moonlighting. The mechanism of how bacterial moonlighting proteins translocate to the cell exterior has remained unknown. They can be released from dead or damaged cells and then bind to neighboring cells, or they can be secreted onto the cell surface by an as-yet-undescribed mechanism [40]. Most of multifunctional proteins described in literature are virulence factors of Gram-positive pathogens. Homologs have been also identified either cell-surface-associated and/or in extracellular space of commensal bacteria including lactobacilli. Their number and moonlighting functions have significantly increased during the last five years. The identified immunoreactive glycolytic enzymes glyceraldehyde-3-phosphate dehydrogenase, phosphoglycerate kinase, glucose-6-phosphate isomerase and 30S ribosomal protein are among the most common of the commensal bacterial moonlighting proteins [41]. Lactic acid bacteria display on the cell surface cytosolic proteins that recognize yeast mannan. The glyceraldehyde 3-phosphate dehydrogenase (GAPDH) is involved in glucose metabolism, catalyzing the metabolism of glucose to release carbon as an energy source, however the other study shown that play important role in adhesion phenomena, or as immunomodulatory factor and recognizes human A and B blood group antigens [42, 43]. Studies have shown that commensal *Lactobacillus crispatus* and several other species of the acidophilus group of *Lactobacillus* have GAPDH as major constituents of their extracellular proteome at neutral pHs and that they are easily released from the cell surface into incubation buffer [44, 45]. The catalytic site in the GAPDH protein was suggested to be involved in the binding to colonic mucin since the binding was inhibited by NAD [46]. Extracellular GAPDH of *L. plantarum* also binds fibronectin [47]. GAPDH and phosphoglycerate kinase were found to be secreted to culture media by *L. rhamnosus* GG [48]. In our work we indicated that GAPDH is at least one of immunogenic protein of *Lactobacillus johnsonii* 142 and immunoreactive protein of *L. rhamnosus* LOCK 0900 and suggested the importance in cross-talk between bacteria and their host. Previously it was shown that GAPDH of *S. agalactiae* is an immunomodulatory protein and that recombinant GAPDH activates T and B cells [49]. The GAPDH of *S. pneumoniae* was reported to be antigenic in humans as well as to elicit protective immune responses in the mouse [50].

A part from immunogenic GAPDH, the main immunoreactive protein 42 kDa of *L. johnsonii* 142 was identified as phosphoglycerate kinase (PGK). This enzyme catalyses the reversible phosphoryl transfer between 1,3-bisphosphoglycerate and ADP to form 3-phosphoglycerate and ATP, and plays a crucial role in the Embden-Meyerhof-Parnas pathway of glycolysis and in gluconeogenesis. This important role is reflected by the enzyme structure that has been highly conserved throughout evolution [51]. It has been shown that PGK isolated from *L. delbrueckii* subsp. *lactis* NCC88 plays a crucial function in regenerating ATP, however in not allosteric enzyme suggesting that is not one of the key enzymes of the energy metabolism regulation of *L. delbrueckii* subsp. *lactis* [52]. In addition, PGK has been recognized as protective antigen against various serotype of *Streptococcus agalactiae* and the hyperimmune sera against phosphoglycerate kinase protect neonatal animals from *S. agalactiae* infection [53]. We identified the most immunoreactive 30 kDa protein of *Lactobacillus paracasei* LOCK 0919 (human origin) as fructose/tagatose-1,6-bisphosphate-aldolase which is the fourth enzyme in glycolysis and catalyses the reversible cleavage of fructose-1, 6-bisphosphate into dihydroxyacetone phosphate and glyceraldehyde 3-phosphate. Aldolase was found in mutanolysin extract of *S. pneumoniae.* Antibodies against aldolase protected against respiratory challenge with *S. pneumoniae* [50]. This study demonstrates for the first time that GAPDH, PGK fructose/tagatose-bisphosphate-aldolase, 30S ribosomal protein, glucose-6-phosphate isomerase and aminopeptidase C from Gram-positive commensal strain not pathogen are able to induce the immune response and elicited antibodies.

All studied nonimmune human sera, not only of adult blood donors, but also from umbilical cord sera, contain antibodies recognizing *Lactobacillus casei* LOCK 0919 derived main protein of 90 kDa. The presence of anti-90 kDa protein antibodies in human sera is unclear and our studies are initiated to solve the question of the biological role of such antibodies.

Interestingly, in our previous study we have shown that *L. johnsonii* 142 isolated from mouse with inflammatory bowel disease (IBD) produced polysaccharide with different structure than *L. johnsonii* 151 isolated from healthy mouse, also reflected in their immunoreactivity [33, 34]. The strains varied also considerably in reactivity of their protein. This could suggest the relation of bacterial antigens synthesized by bacteria colonizing the gut with ongoing inflammatory process, especially surface antigens. The question arises whether resident strains of IBD mice could start the regulation of several proteins or production of surface antigens with specific motifs in their structure, which are absent in antigens of bacteria from the normal gut flora. This also may suggest that the antigens of lactobacilli may have an effect on host responses related to the inflammatory process in IBD.

Comparison of the results showed that there was a great diversity in the immunoreactive proteins amongst the lactobacillus strains, however there were some similarities and consistencies amongst strains both within the species and across the species. In particular it may be of relevance that the presence of GAPDH in mouse and human origin strains were detected as one of the reactive proteins with various sera. However, all identified proteins are common immunogenic proteins that are present in several microorganisms and could well contribute to the known immunomodulatory effect of lactobacilli.

## Conclusions

During the last ten years, the number of applications developed or suggested for *Lactobacillus* has gradually increased [54]. One of the fields currently studied is the development of vaccine based on lactobacilli. It has been already shown that *Lactobacillus* increase the immunogenicity of orally administrated vaccines such as rotavirus [55], polio [56], cholera [57] or influenza [58]. Recently, extensively analyzed is the construction of S-layer protein for use in immunization in man or animals [59–61]. The identification in our study of immunoreactive proteins opens the possibility to use them also as carries of antigens or other medically important molecules, possibly in combinations with immunostimulatory or adhesive molecules.

## Methods

### Microorganisms and growth conditions

The strain of *Lactobacillus johnsonii* 151 was isolated from the intestinal tract of healthy mice housed under SPF conditions, whereas *Lactobacillus johnsonii* 142 was isolated from the intestinal tract of mice with experimentally induced inflammatory bowel disease. The species identification was performed using either PCR with primers for 16S–23S rRNA or rep-PCR using GTG5 primers [33, 34]. *Lactobacillus rhamnosus* LOCK 0900 (formerly *Lactobacillus casei* LOCK900 [62]; patent no. 209988) was isolated from feces of a healthy 26-year-old woman, whereas *Lactobacillus casei* LOCK 0919 (formerly *Lactobacillus paracasei* LOCK919 [63]; patent no. 209986), was originally isolated from a fecal sample collected from healthy 5-year-old boy. Both strains were obtained from the Pure Culture Collection of the Technical University, Lódz, Poland. The species of the *Lactobacillus* genus were identified based on the sequences of genomic markers, such as 16S rRNA, *rpoA*, and *pheS* genes [64].

The strains were stored at −75 °C in MRS broth (Difco) supplemented with 20 % glycerol, and were subcultured twice in MRS broth (Biocorp) under anaerobic conditions at 37 °C before use. The growth of the organism was carried out at 37 °C for 48 h.

Other lactobacilli strains: *L. animalis murinus* 148 (mouse origin) and *L. casei* LOCK0912 (human origin) were used for the preparation of rabbit antisera and were cultivated in the same conditions.

### Protein isolation

Bacterial strains were cultured on MRS broth (Biocorp) for 48 h at 37 °C and centrifuged (6000 g, 4 °C, 20 min) than suspended directly in the buffer for electrophoresis according to Heilmann et al. [65]. Briefly, the cell pellet was resuspended in 1 volume of buffer: 0.5 M Tris-HCl (pH = 6,8)/SDS (0,08 % w/v)/glycerol (20 % v/v)/1 mM β-mercaptoethanol (v/v) and boiled for 5 min. After centrifugation, proteins were precipitated from the resulting supernatant using 3 volumes of cold 95 % ethanol (POCh). After overnight incubation at 4 °C, the precipitated proteins were centrifuged (12 000 rpm) and dissolved in water. Protein concentration was analyzed using the Lowry’s method [66].

In experiments with *Lactobacillus rhamnosus* LOCK900 which is characterized by high amount of slime production [67], before protein isolation procedure, the polysaccharide slime which was loosely attached to bacterial cells was removed mechanically by several centrifugation at 14 500 g.

### SDS-PAGE and immunoblotting

Equal amounts of proteins samples (10 μg) were analyzed on SDS-PAGE using 5 to 12.5 % gels according to Laemmli [68]. Electrophoretic separation were carried out at 100 V, using Tris-Glycine-SDS as running buffer. After electrophoresis, gels were stained with Coomassie Brilliant Blue (Serva) or soaked in transfer buffer (10 mM Tris-HCl, 150 mM glycine, 20 % methanol, pH 8.3) for 30 min. and transferred to a polyvinylidene difluoride membrane (Millipore) for immunoblotting (for 1 h at 100 V). Then the membranes were blocked in phosphate buffered saline (PBS) containing 1 % of bovine serum albumin (BSA, KPL) for 1 h. Afterwards, the membranes were washed three times with PBS containing 0.25 % Tween 20 (Sigma-Aldrich), PBS-T (Institute of Immunology and Experimental Therapy, PAN), and incubated in a selected sera in 1 % BSA for 2 h at 37 °C. We tested different dilutions of sera but the most effective was: for rabbit sera 1:10000, mouse sera 1:1000 and for human sera 1:3000 (reproducible results, without the Hook effect). Next, the membranes were washed three times with PBS-T. After washing, the membranes were incubated for 1 h in alkaline phosphatase-conjugated goat-anti-rabbit/mouse/human IgG antibodies (Sigma) diluted 1 : 5000. Finally, the membranes were washed as six times with PBS-T, and visualized with solution containing nitro blue tetrazolium (NBT, Roth), 5-bromo-4-chloro-3-indolyl phosphate (BCIP, Roth), and MgCl_2_ (POCh) for 5 s. Image acquisition (exposure time 1–4 min) was performed using VersaDoc Imaging System (Bio-Rad).

### Protein identification

After Western blot analysis, the immunoreactive proteins were separated and purified by preparative electrophoresis in denaturing condition (SDS-PAGE) using Prep-Cell apparatus (Model 491 Bio-Rad). Bands of interest were cut out and digested by trypsin (Roche) to obtain a mixture of peptides and analyzed by liquid chromatography (LC), where the mass fragments were measured using mass spectrometer LC-MS/MS Orbitrap (Thermo). Peptides were first trapped and desalted on the enrichment column (Zorbax 300SB-C18, 0.3 × 5 mm, Agilent) for 5 min (solvent: 2.5 % acetonitrile/0.5 % formic acid), then separated on a Zorbax 300SB-C18, 75 μm × 150 mm column (Agilent), using a linear gradient from 10 to 32 % B (solvent A: 5 % acetonitrile in water, solvent B: acetonitrile, both with 0.1 % formic acid). Ions of interest were data-dependently subjected to MS/MS, according to the expected charge state distribution of peptide ions. Proteins were identified by comparative analysis of peptides masses (NCBInr, UniProt, Bethesda, USA), using MS/MS ion search of the Mascot search engine (Matrix Science, London, UK, http://www.matrixscience.com/) and statistical analysis. Only peptide matches with a score of 1000 or above were accepted. Immunoreactive properties of separated proteins were re-tested using immunoblotting.

### Serum samples

Thirteen sera were used: four polyclonal rabbit sera, three mouse sera and six human sera (three of healthy adult volunteers and three from umbilical cord blood). Serum was obtained from rabbits immunized with bacterial mass of *L. paracsei* LOCK 0912 (s1), *L. animalis murinus* 148 (s2), *L. johnsonii* 142 (s3) and *L. johnsonii* 151 (s4) as described before [33]. Briefly, rabbits (5–6 months old) were immunized twice a week with a dried cell mass suspended in PBS. After the first subcutaneous injection with 100 μg/ml, the succeeding doubly increasing amounts (200 to 6400 μg/ml) were injected intravenously. After the last injection rabbits were bled, the separated antisera were decomplemented (56 °C, 30 min) and stored at −20 °C. Preimmune serum was used as a control. The experiments were approved by the 1^st^ Local Committee for Experiments with the Use of Laboratory Animals, Wroclaw, Poland (number 12/2012). Serum was obtained from germ free mouse (GF, s5), mouse kept under specific-pathogen-free conditions (SPF, s6) and form mouse kept under conventional conditions (CV, s7). Germ free BALB/c mice were kept under sterile conditions in Trexler-type plastic isolators, exposed to 12:12-h light-dark cycles and supplied with autoclaved tap water and 50 kGy irradiated sterile pellet diet Altromin 1410 (Altromin, Lage, Germany) ad libitum. Fecal samples were weekly controlled for the presence of aerobic and anaerobic bacteria, molds and yeast by standard microbiological methodology. Conventional BALB/c mice were kept in IVC cages (Tecniplast, Italy), exposed to 12:12-h light-dark cycles and fed with the same sterile diet as GF counterparts. Animal experiments were approved by the committee for protection and use of experimental animals of the Institute of Microbiology. v.v.i., Academy of Sciences of the Czech Republic. Human adults sera were obtained from healthy volunteers (s8-s10) and human umbilical cord sera from healthy women (s11-s13) were obtained from the Obstetric Clinic of the Medical University of Wroclaw. Samples were obtained with patients’ written informed consent. The use of umbilical cord sera samples in this study was approved by the Medical Ethics Committee of the Medical University of Wroclaw (number KB-882-2012) and was conducted in accordance with the Helsinki Declaration of 1975.

## Abbreviations

BCIP: bromo-4-chloro-3-indolyl phosphate
BSA: bovine serum albumin
CV: convetional
EF-Tu: elongation factor Tu
GAPDH: glyceraldehyde 3-phosphate dehydrogenase
GF: germ free
IBD: inflammatory bowel disease
L.: *Lactobacillus*
LC: liquid chromatography
MucBPs: putative mucus binding proteins
NBT: nitro blue tetrazolium
PBS: phosphate buffered saline
PGK: phosphoglycerate kinase
s: serum
SDS-PAGE: sodium dodecyl sulfate – polyacrylamide gel electrophoresis
SPF: specific pathogen free
